# An assessment of the dietary habits among road cyclists competing in amateur races

**DOI:** 10.1002/fsn3.3074

**Published:** 2022-10-21

**Authors:** Ewelina Matusiak‐Wieczorek, Lidia Pyciarz, Marek Drobniewski, Andrzej Borowski

**Affiliations:** ^1^ Health Sciences Department, Sports Medicine Institute Medical University of Lodz Lodz Poland; ^2^ Health Sciences Department Medical University of Lodz Lodz Poland; ^3^ Medical Department, Orthopedics Clinic Medical University of Lodz Lodz Poland

**Keywords:** amateur cyclists, eating habits, fluid intake, hydration, nutrition

## Abstract

From year to year, practicing various sports by amateur athletes is becoming more and more popular. One of such sports is road cycling. To achieve very good sports performance athletes should pay attention not only to physical activity but also to proper nutrition and hydration of the body. The aim of the study was to assess amateur cyclists' dietary habits, especially nutrition and hydration, including assessment of the regularity of eating meals, type of consuming products and fluid intake preferences. The study recruited 41 men aged 23–75 years (43.76 ± 13.25) participating in amateur race road cycling. To obtain information about nutrition and hydration, an original questionnaire was used. Out of all participants, 65.9% declared that they pay attention to their diet, and as many as 75.6% indicated that they eat meals regularly. The vast majority (43.9%) of the cyclists declared consuming four meals a day. Most of the cyclists consumed meat products several times a week—73.2% and dairy products—43.9%. The participants most often chose only one portion of fruit (41.5%) and vegetables (31.7%) during the day. The vast majority of cyclists consumed 3 L of fluids on a training day—51.2%. It turned out that all of the athletes hydrated during training: before it was 68.3% and after training—92.7%. We conclude that the amateur cyclists pay a lot of attention to their nutrition and hydration. During the day, most athletes eat an appropriate amount of meals on a regular basis and drink the right amount of fluids. However, eating of dairy, fruits and vegetables, or hydrating before exercising is insufficient.

## Graphical abstract

The problem of proper nutrition among athletes is common and very important. Recently, there are more and more problems related to nutrition among professional athletes, who have a staff of people working on developing the correct diet for a specific athlete. Taking this into account, we decided to examine the eating habits in the group of amateur athletes, people who do not have easy access to doctors, dieticians, physiotherapists or other specialists who educate how to act in order to achieve good results in sports and maintain a good health.

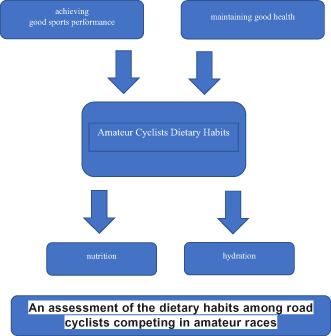

## 1 INTRODUCTION

Road cycling is an endurance sport in which competitors race at different distances, in different climatic conditions and on various routes. The main factors influencing the performance of endurance training are the type, time, and intensity of the exercises, although proper nutrition and hydration are extremely important. Providing the right amount and quality of meals and fluids before, during, and after the race contributes to the improvement of the competitors' efficiency, delays the occurrence of fatigue, and protects against injuries or simply allows an athlete to maintain a good condition (Rothschild et al., 2020).

The optimal diet for achieving peak performance varies from athlete to athlete. An attempt is made to determine the right amount and composition of meals that will be the best for a particular person. Many studies show that the diet should be primarily varied, consisting of lean meat, fish, poultry, fresh vegetables and seasonal fruit (Bean, 2017; Celejowa, 2017; Truswell, 2003). However, it is worthwhile to avoid processed products (Burke, 2007). The basis of proper and healthy nutrition is a balanced consumption of food, consisting of four main components: carbohydrates, proteins, fats, and water (Bean, 2017; Celejowa, 2017; Truswell, 2003). The percentage of each of these components in the athlete's diet significantly affects the quality of training and the race’ performance (Burke, 2007; Griffin, 2014).

It seems obvious that an appropriate selection of food and fluids by athletes, as well as the time of intake throughout the day, provides sufficient energy, replenishment of glycogen stores, maintenance of blood glucose levels, and repair of tissue and helps the improvement of sports performance and the recovery from exercise and exercise‐induced injury. However, there is research which suggests that dietary intake of athletes does not meet sport nutrition recommendations (Muros et al., 2021). What is more, there are reports that competitive athletes eat unhealthily and are at risk of eating disorders (Gorrell et al., 2019; Muros et al., 2020). This may be due to a lack of nutrition knowledge or even the lack of access to this field of knowledge among athletes (Boumosleh et al., 2021; McLeman, 2019; Sparks et al., 2018). If that kind of situation is observed among professional athletes, there is a high probability that it may occur among amateur athletes. This group of athletes does not have a staff of well‐qualified coaches, doctors, and dietitians at their disposal on a daily basis. They also do not always have access to professional literature. This should be re‐emphasized that there is a possibility that amateur athletes do not have enough knowledge about nutrition (Boumosleh et al., 2021; Sparks et al., 2018). That is why we decided to conduct the study in which we tried to know and present dietary habits among amateur road cyclists.

The aim of our study was to determine amateur cyclists':

1. nutrition, including assessment of the regularity of eating meals, type of consuming products, nutritional control and the use of professional counseling,
2. every day hydration and hydration practices before, during, and after physical activity.

## 2 METHODS

The study recruited 41 men aged 23–75, participating in the Gatta Prestige Race in Zdunska Wola, and in the ŻTC (Żyrardów Society of Cyclists) Bike Race in Lyszkowice, organized in 2017. Only amateur adult cycling athletes were included in the study. During the race day, the researcher briefly informed cyclists about the study objectives and procedures and then obtained written consent from those who expressed willingness to participate. Athletes who signed the consent form were then asked to fill out a questionnaire (Appendix S1). All cyclists could complete the questionnaire anonymously and had the option to quit it at any stage. The study did not have to receive an ethical approval because according to Medical University of Lodz Bioethics Committee policy, when the study is not a medical experiment, that kind of document is not necessary.

The questionnaire was created by the author, based on the questionnaires available in publications of other researchers (Durkalec‐Michalski et al., 2011; Kałużny et al., 2016; Kopeć, Nowacka, Klaja, et al., 2013; Szczepańska & Spałkowska, 2012). The questionnaire consisted of three sections: the first section included questions on sociodemographic factors, the second with questions and answer options to determine the amateur cyclist's knowledge regarding every day nutrition and hydration strategies to determine the amateur cyclist's every day nutrition and hydration habits before, during, and after the race, and the last section was about cycling training. In the second section, participants answered the questions of the number and frequency of meal and fluid intake and their preferred type of fluid or food products. The detailed characteristics of the participants are presented in Tables 1 and 2; Figures S1 and S2.

**TABLE 1:** Characteristics of the participants in terms of age, height, body mass, and body mass index (BMI)

	Age (years)	Height (m)	Body mass (kg)	BMI (kg/m^2^)
MIN	20	1.60	62	21
MAX	75	1.84	93	31.10
*x̅*	43.76	1.75	73.39	24.01
SD	13.25	0.05	5.80	1.78

**TABLE 2:** Characteristics of the participants in terms of cycling training (training days per week, training hours per day, years of cycling training)

	*N*	%
Training days		
3 days	7	17.1
4 days	9	22.0
5 days	11	26.8
6 days	9	22.0
7 days	5	12.2
Training hours		
1–1.5 h	7	17.1
2–2.5 h	20	48.8
3–3.5 h	11	26.8
4–4.5 h	2	4.9
>5 h	1	2.4
Years of cycling		
≤1 year	4	9.8
>1 ≤ 5 years	19	46.3
>5 ≤ 10 years	14	34.1
>10 years	4	9.8

The data collected from the questionnaire was analyzed using Statistica software (StatSoft version 13).

## 3 RESULTS

Out of all participants, 65.9% declared that they pay attention to their diet, and as many as 75.6% indicated that they eat meals regularly. However, only 17.1% use nutritional counseling services. The vast majority (43.9%) of the cyclists declared consuming four meals a day, 29.3% three meals, and 24.4%—five meals.

When making a detailed analysis of food products consumed every day by the cyclists, it was noticed that whole grain cereal products (e.g., wholemeal pasta, cereals, brown and wild rice, oatmeal, barley, wholemeal bread) were chosen more frequently than cereal products from refined grains (e.g., white bread, white rice, couscous, semolina, wheat pancakes, white flour products) (46.3% vs. 21.9%). There were no vegetarians among the participants; most of the cyclists consumed meat and fish several times a week—73.2%, and every day—21.9%. During the week, cyclists ate white meat most often (52.1%), red meat less often (32.4%), and fish least often (15.5%). Consumption of dairy products (including eggs) several times a week was declared by as many as 43.9% of athletes, and almost 22% consumed it every day. The questionnaire also included a question about the frequency of consumption products rich in simple sugars (e.g., milk chocolate, cakes, cookies, candies), but the obtained results were not so clear. Almost 30% of respondents indicated answer “1–3 times a month”, while “once a week” and “several times a week” were indicated by 24.4% of respondents each (Table 3).

**TABLE 3:** The types of products and frequency of its consumption by the cyclists

Nutrition habits of the cyclists									
Never		1–3 times in month		Once a week		Several times a week		Every day	
*N*	%	*N*	%	*N*	%	*N*	%	*N*	%
Consumption of whole grain cereal products									
2	4.9	2	4.9	7	17.1	11	26.8	19	46.3
Consumption of cereal products from refined grains									
2	4.9	10	24.4	7	17.1	13	31.7	9	21.9
Consumption of meat (including fish)									
0	0	0	0	2	4.9	30	73.2	9	21.9
Consumption of dairy products (including eggs)									
3	7.3	6	14.6	5	12.3	18	43.9	9	21.9
Consumption of products rich in simple sugars									
5	12.2	12	29.3	10	24.4	10	24.4	4	9.7

Analyzing the consumption of fruit and vegetables, it can be noticed that the respondents most often chose only one portion of fruit (41.5%) and vegetables (31.7%) during the day (Table 4).

**TABLE 4:** The consumption of fruits and vegetables by the cyclists

None		1 portion		2 portions		3 portions		4 portions		5 portions and more	
*N*	%	*N*	%	*N*	%	*N*	%	*N*	%	*N*	%
Consumption of fruit during the day											
2	4.9	17	41.5	11	26.8	5	12.2	5	12.2	1	2.4
Consumption of vegetables during the day											
1	2.4	13	31.7	10	24.4	7	17.1	8	19.5	2	4.9

After analyzing the hydration answers, it turned out that the vast majority of cyclists consumed 3 L of fluids on a training day—51.2%; 2 L of fluids were consumed by 24.4% and 17.1% of athletes consumed 4 L or more. However, there were also cyclists who drink only 1 L of fluids—7.3%. Taking into account the type of fluids, the cyclists most often chose still water—21.3%, quite often tea (18.9%), and coffee (13.4%). It turned out that 68.3% of the cyclists hydrated before (30–60 min) training. However, during training, all of the athletes were drinking on average 571 ml of fluid per hour. After training, 92.7% of the athletes were drinking on average 504 ml fluids per hour (Table 5).

**TABLE 5:** The cyclists' hydration preferences on a training day

	*N*	%
Hydration on a training day		
1 L	3	7.3
2 L	10	24.4
3 L	21	51.2
4 L or more	7	17.1
Type of fluids		
Sparkling water	11	8.7
Still water	27	21.3
Isotonic sports drinks available to buy	12	9.4
Self‐prepared isotonic sports drinks	12	9.4
Fruits or vegetables juices	9	7.1
Fruits or vegetables drinks	3	2.4
Fizzy drinks	5	3.9
Tea	24	18.9
Fruit tea	5	3.9
Coffee	17	13.4
Other	2	1.6

Training hydration	Yes		No		The amount of fluid (ml/h)	
	*N*	%	*N*	%	*x̅*	SD
Before training	28	68.3	13	31.7	496	218
During training	41	100.0	0	0.0	571	187
After training	38	92.7	3	7.3	504	152

## 4 DISCUSSION

Cyclists are athletes who pay a lot of attention to their body weight because it influences how fast they can ride. It is well known that many of them are unsatisfied with their weight and try to reduce it (Hoon et al., 2019). Davis et al. tested 86 cyclists, using Eating Attitudes Test (EAT‐26), a test that detects symptoms, the risk of malnutrition and eating disorders. They showed that compared with professional athletes, amateurs are less likely to have eating disorders (Hale et al., 2011). Analyzing the body mass index (BMI) results of the athletes in our study, we find confirmation of this. In the study group there were no cyclists with the BMI level indicating an underweight. The vast majority (73.2%) was within the normal range.

Although proper nutrition is very important, in our study, 34.1% of cyclists declared that they did not pay attention to their diet. Fortunately, 75.6% ate their meals regularly. This is more than in the study by Kałużny et al. (2016) where only 48% of amateur athletes (including cyclists) ate regularly. According to the principles of proper nutrition, 3–5 meals a day should be eaten, at regular intervals (Celejowa, 2017). Our study showed that 68.3% of athletes consumed 4–5 meals daily. Whereas in Kałużny et al. (2016), it was 37%, and in Durkalec‐Michalski et al. (2011)—74.2%. In the Szczepańska and Spałkowska (2012) study, where volleyball and basketball players were included, it was 52%.

Not only when and how many meals athletes eat but also what they eat is very important. Carbohydrates as a source of energy affect the efficiency of cyclists. They can be delivered to the body when consuming cereal products, such as bread, pasta, and groats. It is recommended to choose whole grain products, not refined ones. Our study showed that the cyclists prefer whole grain products more often (46.3%) than refined in their daily consumption. In the study by Durkalec‐Michalski, 51.6% of amateur athletes, including cyclists, declared daily consumption of wholemeal products (Durkalec‐Michalski et al., 2011). In Kałużny et al. (2016), it was reported by 39% of amateur athletes. Whole wheat bread and/or thick groats daily was consumed by 35% of volleyball and basketball players in the Szczepańska and Spałkowska study (Szczepańska & Spałkowska, 2012). It contrast with the Kopeć, Nowacka, Klaja, et al. (2013) study, where only 5% for each of two subgroups of football players ate wholemeal bread and groats, while 25% for each subgroup ate white bread, noodles, and pasta in everyday consumption. And in another Kopeć, Nowacka, and Polaszczyk (2013) study, where shooting athletes hardly consumed whole grain cereal products and the main source of carbohydrates in their diets were white bread, pasta, and potatoes.

Cyclists' diet should also include fruit and vegetables. In addition to carbohydrates, they also provide large amounts of vitamins and minerals. Bean recommends consuming it even 5–9 times a day (3–5 portions of vegetables and 2–4 portions of fruits; Bean, 2017). In our study, these standards were met in the case of vegetables consumption by 41.5%, and in the case of fruits consumption by 51.2% of cyclists. In the study by Durkalec‐Michalski, as many as 67.7% of amateur athletes declared the consumption of raw fruit and/or vegetables several times a day, and in another form, in at least 3 meals a day (48.4%; Durkalec‐Michalski et al., 2011). In Kałużny et al. (2016) study, vegetables were eaten several times a day by 41% and fruit by 72% of amateur athletes. In the Szczepańska and Spałkowska study, it was respectively 21% and 23% among all the volleyball and basketball players, who consume vegetables and fruits mostly in a raw form (Szczepańska & Spałkowska, 2012). In turn, Sanchez‐Benito and Soriano (2007) found in their study an inadequate supply of vegetables and fruit in the diet of young cyclists.

Considering eating meat, in our study, only 21.9% of cyclists declare everyday consumption. At Kałużny et al. (2016), it was as much as 81% of amateur athletes. It was similar with Szczepańska and Spałkowska—70% of players (Szczepańska & Spałkowska, 2012).

Considering dairy consumption, it has to be pointed out that in our study, we did not ask for milk and dairy consumption but only for dairy. The most common response was dairy consumption several times a week—43.9%. In Durkalec‐Michalski et al. (2011) study, almost 42% of amateur athletes declared everyday consumption of milk and dairy products in at least 2 meals. As many as 77% of amateur athletes surveyed by Kałużny et al. (2016) declared daily consumption of milk and dairy products. In our study, the daily consumption was only 21.9%, which is a relatively low result compared with the above studies.

Some athletes said that they choose sweets as a snack. Unfortunately, these types of products adversely affect the level of glycemia, and the consumption of it may lead to an increase in body weight, which is not desired in cycling (Celejowa, 2017). In our study, many cyclists declared the consumption of products rich in simple sugars once a week—24.4%, and even several times a week (also 24.4%). Only 12.2% of respondents do not eat sweets at all. In the study by Durkalec‐Michalski et al. (2011), daily consumption of sweets was declared by 12.9% of athletes, and 29% of athletes completely resigned from this type of snacks.

In addition to proper nutrition, adequate hydration is essential for the good health and performance of an athlete. In our study, the vast majority of cyclists consumed two or more liters of fluid during the day, the most common answer was 3 L—51.2%. In the Szczepańska and Spałkowska study, it was 2–2.5 L (27%; Szczepańska & Spałkowska, 2012). In the study by Kałużny et al., 69% of athletes declared the consumption of at least 2.5 L of fluid a day. However, as many as 25% of athletes preferred carbonated drinks, which are not recommended as a hydration fluid (Kałużny et al., 2016). We also asked about the most frequently chosen fluids (It was possible to give more than one answer). That is why we know that in our study group, carbonated drinks were used much less often—12.5%. The most frequently chosen fluid was still water—65.9% and tea—58.5%. In Szczepańska and Spałkowska mineral water (68%) and juices (17%) were chosen (Szczepańska & Spałkowska, 2012).

Talking about fluid intake in the context of physical effort, it is very important to pay attention to hydration not only during, but also before and after training. As Bean recommended, about 500 ml of fluid should be consumed 2 h before training. During training, about 150–300 ml should be drunk regularly every 15–20 min. In turn, after training, the loss of fluids should be replaced by drinking 450–675 ml for every 0.5 kg of lost body weight (Bean, 2017).

In our study, the cyclists showed great awareness of the issue of body hydration. All of them declared drinking fluids during training, in the average amount of 571 ml/h. Not much less, because 92.7% of respondents hydrated after their training, drinking on average of 504 ml/h. The smallest number of athletes—68.8% consumed fluids before starting physical activity, delivering on average of 496 ml/h. Similar results were obtained by Kałużny et al., where also 92% of respondents declared hydration after training. However, only 83% drank during exercising. There is no information in the study about hydration before training. It is also not possible to compare the amount of fluids consumed because the authors did not ask about it (Kałużny et al., 2016). Kunces et al. asked about the amount of fluids consumed during exercise. In their study, 20 men training in amateur cycling declared on average consumption of 863 ml of fluids per hour. Information was collected on the day of the 162 km race. As researchers stated, it was a hot day, which could have had an impact on the amount of the athletes' fluid intake. Unfortunately, the study did not include data on hydration before and after the race, which in such conditions was certainly an important factor influencing the performance and sports results of competitors (Kunces et al., 2016).

To sum up, we decided to conduct the study because we were interested in nutrition and hydration habits among amateur road cyclists. We chose this group of athletes because we have found a lot of research on the problem of nutrition and hydration among professional athletes, practicing various sports. However, to our knowledge, there is a little research analyzing this problem among amateur road cyclists. To implement our assumptions, we managed to reach the cyclists taking part in amateur races organized in Poland. After briefly familiarizing the participants of the races with the course of our study, we were finally able to collect data from 41 cyclists. We realize that the small number of participants is the basic limitation of this study and makes it difficult to generalize results. What is more, we are aware that participants included to this study were in a wide range of age, some of them were living in a villages or in smaller or bigger cities, they were in different professional situations, and what is the most important, there were differences in their cycling trainings. However, some similarities and repetitive patterns can be found in the studied amateur cyclists' behaviors and preferences, which is why we decided to conduct this study and determine the eating habits in this group of athletes. The differences mentioned above certainly affect food preferences and may bias the study's results. However, we trust that our study provides some insight into the nutrition and hydration situation among amateur cyclists and may well be an introduction to further research carried out on this topic, in a more homogenous and bigger group of amateur athletes.

To collect data, we used an original questionnaire, which is a subjective research tool and may bias the results. On the other hand, questionnaires are one of the cheapest, easy‐to‐use tools that can be widely used among different populations.

We would like to suggest that further research on this topic, should be for example carried out between amateur and professional road cyclist athletes, because it will give an opportunity to compare obtained results and make an interesting statistical analysis.

Based on the research carried out and the available literature, it can be concluded that the frequency of appropriate eating behavior among amateur road cycling practitioners is quite high. The vast majority of athletes consume an adequate number of meals during the day on a regular basis. A large number of cyclists pay attention to what they eat. Unfortunately, only a few take advantage of professional nutrition counseling.

Amateur cyclists make some nutritional mistakes. That is why, it is necessary to introduce changes in the diet of these athletes. These changes should include increasing the consumption of dairy products, fruits and vegetables, and reducing the consumption of meat and foods rich in simple sugars.

Considering hydration, most amateur road cyclists have good fluid intake habits, both in type and amount. However, awareness of the need to hydrate the body before training should be increased.

To sum up, there is a great demand for education and awareness‐raising in the field of proper nutrition and hydration among amateur road cyclists. Therefore, further research should be conducted to analyze the eating habits in this group of athletes.

## ACKNOWLEDGMENTS

The authors are responsible for the content and writing of the paper.

## CONFLICT OF INTERESTS

The authors have no conflict of interest.

## ETHICS STATEMENT

According to Medical University of Lodz Bioethics Committee policy, when the study is based on surveys and does not pose any threats to the participants, ethical approval is not necessary. That is why this study did not require formal research ethics approval.

## Supporting information


Figure S1

Appendix S1

## Data Availability

The data that support the findings of this study are available from the corresponding author upon reasonable request.
