# Supplementary material for: An assessment of the dietary habits among road cyclists competing in amateur races
Source: Food Sci Nutr. 2022 Oct 21;11(1):428–33. doi: 10.1002/fsn3.3074 (PMC9834813; doi:10.1002/fsn3.3074)
Supplement: Supplementary file 1 — Figure S1 [file FSN3-11-428-s001.docx]

Figure S1 Percentage characteristics of the participants in terms of living place.

Figure S2 Percentage characteristics of the participants in terms of professional situation.
