# Supplementary material for: An assessment of the dietary habits among road cyclists competing in amateur races
Source: Food Sci Nutr. 2022 Oct 21;11(1):428–33. doi: 10.1002/fsn3.3074 (PMC9834813; doi:10.1002/fsn3.3074)
Supplement: Supplementary file 2 — Appendix S1 [file FSN3-11-428-s002.pdf]

participant number …………..

Part 1. General information

1. Age (years)………………………….
2. Body mass (kg): …………………
3. Height (cm): …………………..

4. Place of living:
a) village
b) city < 20 thous. inhabitants
c) city > 20 < 100 thous. inhabitants
d) city > 100 < 500 thous. inhabitants
e) city > 500 thous. inhabitants

5. Professional situation
a) student
b) professionally active person
c) retired person
d) pensioner

Part 2. Information on eating habits

6. Do you pay attention to your diet?
a) YES
b) NO

7. How many meals do you eat during the day?………………………………

8. Do you eat your meals regularly?
a) YES
b) NO

9. Do you ever snack between meals? (you can mark several)
a) YES, fruits or vegetables
b) YES, sandwiches
c) YES, sweets
d) YES, salty snacks
e) YES, others: ……………………………………………………………………
f) NO

10. How often do you eat sugar-rich foods (eg milk chocolate, cakes, cookies, candies)?
a) never
b) 1 - 3 times a month
c) once a week
d) several times a week
e) every day

11. How often do you eat whole grain cereal products (eg, wholemeal pasta, cereals, brown and wild rice, oatmeal, barley, wholemeal bread)?
a) never
b) 1 - 3 times a month
c) once a week
d) several times a week
e) every day

12. How often do you eat refined grains (e.g. white bread, white rice, couscous, semolina, wheat pancakes, white flour products)?
a) never
b) 1 - 3 times a month
c) once a week
d) several times a week
e) every day

13. How many portions of fruit do you eat during the day (portion e.g. one apple, half a glass of blueberries, etc.)?
a) none
b) 1 portion
c) 2 portions
d) 3 portions
e) 4 portions
f) 5 and more portions

14. How many portions of vegetables do you eat during the day (portion e.g. one tomato, small cucumber, etc.)?
a) none
b) 1 portion
c) 2 portions
d) 3 portions
e) 4 portions
f) 5 and more portions

15. How often do you eat meat and fish?
a) never
b) 1 - 3 times a month
c) once a week
d) several times a week
e) every day

16. What meat do you eat most during the week? (you can mark more than one)
a) white (chicken, turkey, rabbit)
b) red (pork, beef, lamb)
c) fishes

17. How often do you eat fish dishes?
a) never
b) 1 - 3 times a month
c) once a week
d) several times a week
e) every day

18. How often do you eat dairy and dairy products? (including eggs)
a) never (please go to question 20)
b) 1 - 3 times a month
c) once a week
d) several times a week
e) every day

19. How many eggs do you eat in a week?
a) one or less a week
b) two a week
c) three a week
d) four or more a week

20. How many liters of fluid do you consume per day during the training period?
a) less than 1litre
b) 1 litre
c) 2 litres
d) 3 litres
e) 4 or more litres

21. What kind of drinks do you choose most often? (you can mark more than one)
a) sparkling water
b) still water
c) isotonic sports drinks available to buy (Oshee, Powerade, etc.)
d) self-prepared isotonic sports drinks
e) friuts or vegetables juices
f) friuts or vegetables drinks
g) fizzy drinks
h) tea
i) friut tea
j) coffee
k) others:...

22. Do you hydrate before (30-60 minutes) training?
   a) NO
   b) YES (in what quantity …....................................ml,
      at what time...)

23. Do you hydrate during training?
   a) NO
   b) YES (in what quantity …....................................ml,
      at what time...)

24. Do you hydrate after training?
    a) NO
    b) YES (in what quantity …....................................ml,
       at what time..)

25. Do you use nutritional or dietary counseling (for example, a dietary clinic, trainer, science books, courses or training, thematic magazines)?
    a) NO
    b) YES. What is it?
       ………………………………………………………………………………

PART 3. Information about cycling training

1. How many days a week do you train?
   ………………………………………………..
2. How many hours do you train in one day?
   ………………………………………………..
3. How many years have you been training cycling?
   ……………………………………………
